# Analysis of long term CD4+CD25highCD127- T-reg cells kinetics in peripheral blood of lung transplant recipients

**DOI:** 10.1186/s12890-017-0446-y

**Published:** 2017-07-18

**Authors:** Davide Piloni, Monica Morosini, Sara Magni, Alice Balderacchi, Luigia Scudeller, Emanuela Cova, Tiberio Oggionni, Giulia Stella, Carmine Tinelli, Filippo Antonacci, Andrea Maria D’Armini, Federica Meloni

**Affiliations:** 1 Department of Internal Medicine, University of Pavia, Pavia, Italy; 2 Cardiothoracic and Vascular Department, Pneumology Unit, IRCCS Policlinico San Matteo Foundation, Pavia, Italy; 3 Clinical Epidemiology and Biometry Unit, Scientific Direction, IRCCS Policlinico San Matteo Foundation, Pavia, Italy; 4 Cardiothoracic and Vascular Department, Cardiac Surgery Unit, IRCCS Policlinico San Matteo Foundation, Pavia, Italy

**Keywords:** Lung transplantation, Immunology, T-regulatory cell, Long term follow-up

## Abstract

**Background:**

The role of CD4^+^CD25^high^CD127^−^ T-reg cells in solid-organ Transplant (Tx) acceptance has been extensively studied. In previous studies on kidney and liver recipients, peripheral T-reg cell counts were associated to graft survival, while in lung Tx, there is limited evidence for similar findings. This study aims to analyze long term peripheral kinetics of T-reg-cells in a cohort of lung recipients and tests its association to several clinical variables.

**Methods:**

From jan 2009 to dec 2014, 137 lung Tx recipients were submitted to an immunological follow up (median: 105.9 months (6.7–310.5)). Immunological follow up consisted of a complete blood peripheral immuno-phenotype, inclusive of CD4^+^CD25^high^CD127^−^ T and FOXP3+ cells. We tested the association between T-reg and relevant variables by linear OR regression models for repeated measures, adjusting for time from Tx. Also, by ordered logistic models for panel data, the association between Chronic Lung Allograft Dysfuncton (CLAD) onset/progression and T-reg counts in the previous 3 months was tested.

**Results:**

Among all variables analyzed at multivariate analysis: Bronchiolitis Obliterans Syndrome (OR −6.51, *p* < 0.001), Restrictive Allograft Syndrome (OR −5.19, *p* = 0.04) and Extracorporeal photopheresis (OR −5.65, *p* < 0.001) were significantly associated to T-reg cell. T-reg cell counts progressively decreased according to the severity of CLAD. Furthermore, patients with higher mean T-reg counts in a trimester had a significantly lower risk (OR 0.97, *p* = 0.012) of presenting CLAD or progressing in the graft dysfunction in the following trimester.

**Conclusions:**

Our present data confirm animal observations on the possible role of T-reg in the evolution of CLAD.

## Background

Lung transplant (Tx) is the only therapy for patients with end-stage respiratory failure, but despite the use of potent immunosuppressive protocols, the long-term survival of lung graft is hampered by the occurrence of chronic lung allograft dysfunction that occurs in nearly 50% of patients by the 5th post-Tx year [1]. According to recent classification, there are two major clinical phenotypes of chronic irreversible allograft dysfunction (CLAD): the so called obstructive form: bronchiolitis obliterans syndrome (BOS), (nearly 70% of cases) [2], and the newly described restrictive allograft syndrome (RAS) [3, 4]. CLAD is considered a complex multifactorial process [5], which leads to chronic airway inflammation and tissue injuries and ultimates in a fibro-reparative response. This can involve either the airway lumen in BOS, or the interstitial and airways in RAS [6]. Although recent evidence ascribes an important pathogenic role in CLAD to aspecific inflammatory mechanisms [7–10], the importance of specific immunity can’t be denied. Several previous studies support a crucial role for allo and auto-reactive T cells in BOS pathogenesis [11–14]. T cell response as well as the production of allo or auto-antibodies have been described, and are dependent on the migration of antigen-presenting cells in secondary lymphoid organs and on the direct stimulation of T cells within graft [15, 16]. All of this experimental and clinical evidence strongly points out the difficulties in achieving immunologic tolerance, either central or peripheral, of lung graft [17–19].

Since 1995, when Sakaguchi described a CD4^+^CD25^+^T cell subset displaying regulatory properties, several subsets of T-reg cells have been described and classified as natural T-reg, thymus-derived and naturally committed to immunoregulation, and inducible T-reg, generated in the periphery during immune response. So far the best characterised T-reg population is the thymus-derived CD4 subset, constitutively expressing CD25 (at a high rate) and FoxP3, a transcription regulator which controls the maturation and function of T-reg cells. Although a specific and exclusive marker of T-reg activity has not yet been identified, the diminished surface expression of CD127, as well as the expression of CD39 or CD152, are considered characteristic of CD4^+^CD25^+^ T-reg cells. [20, 21]These molecules can be expressed, even if transiently, as Foxp3, by other cell types including recently activated effector T cells. However, experimental evidence suggests that most of the Foxp3+ T-reg cells are within the CD4^+^CD25^high^CD127^−^ cell population, so that sorting of this subset is actually considered the best method for the isolation and in vitro amplification of these cells [22].

The role of CD4^+^CD25^high^CD127^−^ T-reg cells in Tx acceptance has been studied in experimental models and also in clinical settings. Animal studies demonstrated that CD4^+^CD25^+^ Foxp3+ T-reg cells are expanded in tolerized animals, that tolerance can be infectiously transferred to naïve animals, and appears to be a specific and localized phenomenon. These data have been partially confirmed in kidney and liver Tx recipients, showing a positive correlation between graft survival and the number of circulating CD4^+^CD25^+^ T-reg cells, as well as a correlation between their peripheral fluctuation and the occurrence of acute and chronic rejection [18, 23, 24].

As for lung Tx, evidence is limited and somewhat contradictory [25]. In previous cross- sectional studies, we showed that lung Tx recipients with BOS had significantly lower peripheral CD4^+^CD25^high^ T-reg cells than clinically stable lung recipients, and demonstrated their functional regulatory profile, in vitro [26, 27]but subsequesnt studies failed to demonstrate a correlation between T-reg cell counts and long term lung Tx outcome [28–30].

To date, however, little is known about the long term evolution of peripheral CD4^+^CD25^high^CD127^−^ regulatory T cells in lung Tx. The present study aims at analyzing their long-term kinetic on a cohort of lung recipients and at testing its association with several clinical and pharmacological variables (CLAD, treatment, infections, kidney dysfunction or neoplasia).

## Methods

### Patients

In our Center, LTx has been performed since 1991. The study was conducted from 1st January 2009 to 31st December 2014. During this period 137 lung recipients entered the immunological follow-up, with a median follow-up of 105.9 months (range 6,7–310,5). All patients were submitted to the assessment of a complete peripheral immune phenotype at least twice a year.

Our immune suppression protocol has undergone some changes over time. No patients underwent induction treatment at time of transplantation. All patients transplanted between 2001 and 2007 were treated with a triple immunosuppressive regimen including cyclosporine, azathioprine, and prednisone, whereas patients transplanted since January 2007 received a modified standard triple regimen, with tacrolimus, mycophenolate mofetil, and prednisone. In case of refractory acute rejection (AR), patients were switched from cyclosporine to tacrolimus and from azathioprine to mycophenolate mofetil. In the presence of documented renal dysfunction, patients were treated with low- dose tacrolimus plus everolimus.

All patients underwent surveillance and on-need bronchoscopies; biopsy-proven episodes of AR following criteria [31] were treated with steroid boluses and, in case of AR recurrence or persistence, with a standard anti-thymoglobulin course and a modulation of the IS regimen. Our surveillance protocol has been reported in previous studies [32]. BOS diagnosis and severity grades has been assessed according to published guidelines [6, 33–35]. The CLAD subtype RAS has been retrospectively re-classified for patients diagnosed before 2013, according to radiological (CT scan showing a pattern of persistent interstitial/upper lobe fibrosis) and functional criteria (persistent decline in forced expiratory volume in 1 s (FEV 1) of >20% compared to the best post Tx value and a decline in total lung capacity of >10% compared to baseline) [33–35]. In case of a BOS 0p or early RAS diagnosis, patients were prescribed a 3-month course of chronic low-dose azithromycin. At the same time, patients underwent gastro-esophageal reflux assessment and maximization of anti-reflux medical treatment. In case of a further decline consistent with a CLAD diagnosis, since 2003, patients are referred to the Apheresis Unit for compassionate ECP treatment [36]. Severity of CLAD has been graduated according to the degree of functional impairment as described for BOS [6, 33–35]. Our cytomegalovirus surveillance protocol has been detailed in previous studies [37].

The ethics review committee of the IRCCS Policlinico San Matteo of Pavia approved the research n° ICS 30.4/RF00.65.

### Flow cytometric determination of peripheral lymphocyte subsets

Flow cytometry was performed on a Beckman Coulter Navios using Kaluza software (Beckman Coulter). Briefly, 50 μl of fresh whole blood was incubated with the appropriate amounts of fluorochrome-labeled monoclonal antibodies CD45 APC Alexa Fluor 750 (Allophycocyanin-Alexa Fluor 750; clone J33), CD4 APC (Allophycocyanin; clone 13B8.2), CD69ECD (R Phycoerythrin-Texas Red; clone TP1.55.3), CD25 PE (R Phycoerythrin; clone B1.49.9), CD127 PC5 (R Phycoerythrin-Cyanine 5.1; clone R34.34) for the T-reg and CD45 APC Alexa Fluor 750, CD4 APC, CD3 FITC (Fluorescein isothiocyanate; clone UCHT1), CD8 PE (R Phycoerythrin; clone B9.11), CD56 PC5 (; clone N901), CD16 PC5 (PC5.1; clone 3G8), CD19 PC7 (PC 7; clone J3–119) for the lymphocytic population (Beckman Coulter) at room temperature in the dark for 15 min using appropriate mouse immunoglobulin isotypes as a control. Following incubation, 1 ml erythrocyte lysing solution (VersaLyse, Beckman Coulter) was added to the samples and incubated under the same conditions for 20 min. In some samples, peripheral blood mononuclear cells (PBMC) were stained with CD4 APC, CD25 FITC (clone B1.49.9), and CD127 Alexa Fluor 647 (clone HIL-7R-M21) (BD Pharmingen), fixed and permeabilized, followed by intracellular staining with Foxp3 PE (clone PCH101) or control IgG1 (Human Regulatory T cell Staining kit, eBioscience) for 30 min. Finally, the cells were characterized by flow cytometry analysis.

### T-reg cell analysis

Peripheral blood assessment of regulatory T cell subsets has significantly evolved in the last years, even more so during the realization of this study. For this reason, we started the protocol performing the analysis of peripheral CD4^+^CD25^high^CD127^−^ cell subset, and later on we also started to quantify FOXP3+ cells.

**Fig. 1:**
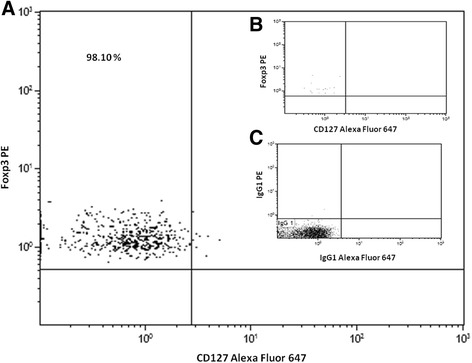
**a**. Representative dot plot of intracellular staining Foxp3 in CD4 + CD25highCD127 - T-reg cell.**b.** Representative dot plot of intracellular staining Foxp3 in CD4 + CD25-CD127pos (0%) T-reg cell. **c.** Representative dot plot of isotope control

Therefore, we could determine that peripheral CD4^+^CD25^high^CD127^−^ T-reg cell subset included a mean of 93.15% (±4.34) FOXP3+ cells (Fig. 1). We also found a high significant correlation between peripheral CD4^+^CD25^high^CD127^−^ T-reg cell counts and FOXP3+ cell counts over the whole cohort. For this reason, and in accordance with published evidence [20] we included CD4^+^CD25^high^CD127^−^ cell determinations in the final statistical analysis, expressed this subset as absolute number (n°/μl peripheral blood) and named these cells approvedCD4^+^CD25^high^CD127^−^ T-reg cells. Analysis performed with CD4^+^CD25^high^CD127^−^ T-reg cells expressed as percentage of the whole CD4 subset, gave analogous results.

### Statistical analysis

Descriptive statistics were produced for demographic, clinical and laboratory characteristics of cases. Mean and standard deviation (SD) are shown for normally distributed variables, and median and interquartile range (IQR) for non-normally distributed variables, numbers and percentages for categorical variables.

The association between T-reg and a number of predictors was explored by means of bivariate and multivariate linear mixed models, with patient and time since Tx as random effects and predictors and time (also) as fixed effects. Clinical and Immunological variables that were included in the analysis are shown in Table 1.

**Table 1:** demographic and clinical features of the patients enrolled in this study

	N° of patients = 137
Mean age at Tx (years ± SD)	46.1 ± 12.86
Sex (M:F)	91:46
Lenght of post-Tx follow-up (months/range, median)	105.9 / 6.7–310.5
*Tx Indications*	
Emphysema / Alpha1 antitrypsin Deficiency	27
Primary graft disfunction	4
Bronchiectasis / Cystic Fibrosis	26
Interstitial lung disease	52
Pulmonary hypertension / Ebstein’s disease/Eisenmenger Syndrome / Mounier-Kuhn Syndrome	25
Rare pulmonary conditions	3
*Type of Tx*	
Single lung Tx	46
Double lung Tx	83
Heart and Lung Tx	8
*Immunosuppression therapy*	
Cyclosporine	23
Tacrolimus	119
Azathioprine	15
Mycophenolate mofetil	88
Rapamycin	37
Prednisone	137
Extracorporeal photopheresis (ever)	39
Azitromycin (ever)	98
	Total of determination = 1943
Stable	796
CLAD	1147
BOS	838
RAS	309
BOS 0p	320
CLAD 1	412
CLAD 2	169
CLAD 3	246

*CLAD* chronic lung allograft dysfunction, *BOS* bronchiolitis obliterans syndrome, *RAS* restrictive allograft syndrome. BOS 0p according to published guidelines [6, 30–32] CLAD 1: includes both BOS1 and RAS patients with FEV1 80–65% + FVC < 80%; CLAD 2: includes both BOS2 and RAS patients with FEV1 64–50% + FVC < 80%; CLAD 3: includes both BOS3 and RAS patients with FEV1 < 50% + FVC < 80%

To assess whether CD4^+^CD25^high^CD127^−^ T-reg cell count was associated to CLAD occurrence or higher CLAD grade in the subsequent 3 months, we fit ordered logistic models for panel data. When a dependent variable has more than two categories and the values of each category have a meaningful sequential order where a value is indeed ‘higher’ than the previous one, then we can use ordinal logistic regression. In this instance, we are comparing patients in CLAD grade > k versus ≤k. The interpretation is: for a one unit change in the predictor variable (e.g. T-reg cell count in previous trimester), likelihood of having CLAD grade > k instead of ≤k is an OR times higher.

In all cases, tests were two-tailed, and the *p*-value cut-off for significance was set at 0.05.

Stata computer software version 14.0 (Stata Corporation, 4905 Lakeway Drive, College Station, Texas 77,845, USA) was used for statistical analysis.

## Results

### Patients

Overall 137 patients were included in this retrospective study and followed-up for a median of 105.9 months (6.7–310.5). Demographics and clinical features of included patients are listed in Table 1, including gender, age at Tx, Tx indication, type of Tx, length of follow-up and type of immunosuppressive drugs used. Some patients (27%) were enrolled in the immunological follow- up at time of Tx, while the others entered the study later in the FU period (median follow-up months at first determination in the latter group: 82,4 months, range 14,0–275,7) this, as stated above, was considered in the statistical analysis.

Being a prospective immunological FU, the overall number of included samples is high: n° 1943 with a median of 14 sample/patient.

### Variables associated to T-reg cell counts

**Fig. 2:**
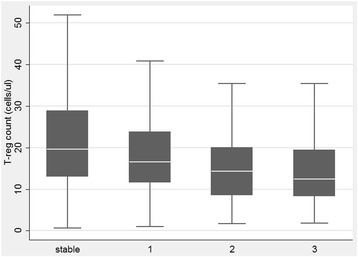
Relation between T-reg cells count and BOS grade (calculated according to BOS severity classification [30]). Figure is purely descriptive. Median, IQR and min/max are depicted. *p < 0.001; §*p* = 0.01

**Fig. 3:**
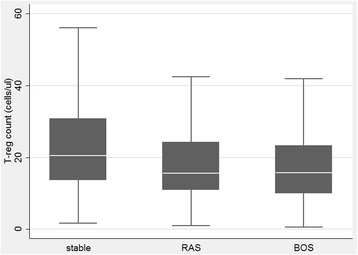
Relation between T-reg cells count and stable, BOS (*p* < 0.001) or RAS (*p* = 0.01) patients’ (BOS was diagnosed according to published guidelines [6, 30–32]. RAS has been retrospectively re-classified according to radiological and functional criteria [30–32]).). Figure is purely descriptive. Median, IQR and min/max are depicted

Results of bivariate and multivariate analysis are shown in Table 2. As for acute rejection, the limited number of samples obtained during an episode of acute cellular (Agrade ≥2, any B grade) or humoral rejection (globally < than 15) did not allow us to assess any statistical association with peripheral T-reg cell number**.** All tested immunological variables (CD3+,CD4+, CD8+, CD19+ and CD16 + CD56+ cells) resulted positively correlated associated to T-reg (Table 2) at the initial bivariate analysis. Among clinical variables only the presence of CLAD, treatment with azathioprine and ECP were significantly associated to peripheral CD4^+^CD25^high^CD127^−^ T-reg cell counts. Given the lack of association with T-reg cell counts with the majority of tested variables only azathioprine, ECP, and CLAD, as well as BOS or RAS and CLAD severity grades were included in the multivariate model. At multivariate analysis, only the association with azathioprine did not retain its significance (Table 2). Furthermore, when BOS severity grade was considered, a significant progression of CD4^+^CD25^high^CD127^−^ T-reg cell decline was observed (Fig. 2). Of note, no difference was detectable between the 2 CLAD phenotypes, BOS and RAS (Fig. 3). The significant negative association of T-reg cell counts with ECP was confounded on the presence and severity of CLAD, and thus was not considered clinically relevant.

**Table 2:** bi- and multi-variate linear regression analysis per CD4^+^CD25^high^CD127^−^

			Bivariate			Multivariate	
Variable	Category	Coef	95% CI	*P*-value	Coef	95% CI	*P*-value
Time	Per each month since Tx	0.16	0.10 to 0.22	**<0.001**	0.18	0.12 to 0.24	**<0.001**
CLAD	RAS	−6.60	−11.594 to-1.603	**0.01**	−5.19	−10.2 to −0.16	**0.04**
	BOS	−8.14	−11.807 to −4.473	**<0.001**	−6.51	−10.26 to −2.77	**0.001**
BOS grade	0p	−2.05	−4.165 to 0.069	0.06			
	1	−2.53	−5.122 to 0.068	0.06			
	2	−6.49	−9.724 to −3.261	**<0.001**			
	3	−4.90	−8.731 to −1.066	**0.01**			
Lymphocytic population	cd3	0.009	0.008 to 0.01	**<0.001**			
	cd 3 cd4	0.021	0.019 to 0.023	**<0.001**			
	cd 3 cd8	0.01	0.008 to 0.011	**<0.001**			
	cd19	0.05	0.038 to 0.057	**<0.001**			
	cd1 6 cd56	0.01	0.005 to 0.008	**<0.001**			
	cd4cd25high	0.60	0.585 to 0.62	**<0.001**			
Immunosuppressive therapy	Cyclosporine	0.20	−3.598 to 4.007	0.92			
	Tacrolimus	−2.71	−5.783 to 0.367	0.08			
	Azathioprine	4.65	0.438 to 8.856	**0.03**	−2.12	−6.73 to 2.48	0.336
	Mycophenolate	0.04	−1.735 to 1.823	0.96			
	Rapamycin	−0.75	−3.202 to 1.703	0.55			
	Prednisone	−6.32	−15.329 to 2.687	0.17			
Azithromycin therapy		−0.74	−2.505 to 1.016	0.41	0.58	−1.28 to 2.43	0.542
Extracorporeal photopheresis		−6.03	−8.259 to −3.804	**<0.001**	−5.65	−8.05 to −3.25	**<0.001**
Kidney failure		−1.36	−3.62 to 0.907	0.24			

Bold characters mean statistically significant variable

Linear mixed models were fitted, with patient and time since Tx as random effects, and individual predictors and time as fixed effects

*CLAD* chronic lung allograft dysfunction, *RAS* restrictive allograft syndrome, *BOS* bronchiolitis obliterans syndrome

### Prediction of CLAD

In patients with higher mean peripheral CD4^+^CD25^high^CD127^−^ T-reg counts, the risk of presenting CLAD or progressing in the graft dysfunction in the subsequent trimester was significantly lower (OR 0.97, 95%CI 0.95–0.99, *p* = 0.012). Variables were included in the model if clinically relevant or statistically significant (at the 0.05 level) at bivariate analysis, without further selection (Table 3).

**Table 3:** ordered logistic regression analysis for association between CLAD grade and a number of predictors, including T-reg in the previous trimester

CLAD grade	OR	*P*-value	95% Conf. interval
CD4 + CD25highCD127-	0.97	0.012	0.95 to 0.99
Azithromycin therapy	5.80	<0.001	2.81 to 11.99
Prednisone	0.22	<0.001	0.13 to 0.39
Trimester	1.01	<0.001	1.01 to 1.02

NB in ordered logistic regression models, OR > 1 implies higher risk of being in a higher category (in this case: CLAD grade, calculated for all CLAD patients according to BOS severity classification [30]) rather than in any of the lower categories; an OR <1, implies a lower risk

## Discussion

On the basis of our results we can confirm and extend our previous observation on the role of CD4 + CD25high T-reg cells in lung graft acceptance/rejection [27]. To our knowledge, this is the first longitudinal study reporting the long-term kinetics of peripheral CD4^+^CD25^high^CD127^−^ T-reg cells in lung recipients, and the first observation, that variation of peripheral T-reg cell counts can predict CLAD onset/progression.

Although, during the study period, a methodological evolution in the identification of T-reg cells subsets occurred, we clearly demonstrated that CD4^+^CD25^high^CD127^−^ subset is significantly enriched with FOXP3+ cells, thus inferring that it is endowed with regulatory functions. Based on these observations, we have referred to these cells as CD4^+^CD25^high^CD127^−^ T-reg cells.

Experimental evidence in animals have strongly suggested that lung graft acceptance is associated to an intra-graft [16, 38] and peripheral [39] T-reg cell expansion and that adoptive transfer of “in vitro” generated and expanded T-reg cells attenuates airway obliteration in a rat OB model [40].

Conversely, human studies have provided, to date, sub-optimal evidence on the protective role of T-reg cells towards chronic graft rejection. Most studies, in fact, have explored peripheral CD4 + CD25+ T-reg cell counts early after Tx, suggesting that a low number of these effectors might predict subclinical or clinical acute kidney rejection [41, 42], and can be significantly affected by the type of immunosuppressive regimen [43, 44]. A few studies are available on lung Tx recipients with conflicting results. Neujahr and colleagues found higher BAL CD4 + CD25high cell counts in patients with biopsy-proven AR, but failed to find a correlation with peripheral blood findings [45]. On the contrary, lower CD4 + CD25high T cell counts were detected both at peripheral and BAL level of BOS patients with respect to stable lung recipients [27, 46].Moreover, an increase in BAL CCR7(+) T-reg cell percentage was found to correlate to a reduced risk of BOS by Gregson and colleagues [47]. Krustrup and colleagues, analyzing CD4 + CD25 + FOXP3+ cell at tissue level, found higher frequencies during acute rejection episodes [29], but failed to demonstrate an association between these cells and the risk of CLAD occurrence [30]. We believe that prediction studies on peripheral T-reg in Tx recipients are invalidated by the high fluctuation of blood-T-reg cell in time, possibly in relation to a number of confounding variables including drugs, infectious complications, neoplasia, kidney failure and ECP treatment.

Thanks to the large number of determinations that we included in this long term study, we took into account this variability in the analysis, thus we succeeded in demonstrating that: 1) peripheral counts of CD4^+^CD25^high^CD127^−^ T-reg cells significantly decrease in CLAD patients; 2) the degree of their decrease is associated with the severity of BOS and, most noteworthy; 3) CLAD onset or GRADE is significantly associated to mean T-reg cell counts in the previous trimester.

Finally, we also tested the association of CD4^+^CD25^high^CD127^−^ T-reg cell counts with a number of clinical variables, including also infections and different immunosuppressive drugs. Interestingly, only azathioprine and ECP were found to be significantly associated to them at the univariate analysis, while, in the multivariate model, only the ECP effect was significant. However, the ECP effect was confounded on CLAD and CLAD severity, and is therefore to be considered irrelevant from the clinical point of view.

Interestingly, unlike previous evidence in literature [26, 48]we could not detect any significant variation of T-reg cell counts with respect to other specific immunosuppressive drugs such as cyclosporine A, tacrolimus or everolimus. The effect of maintenance drugs on T-reg cell counts has been poorly evaluated in humans, the role of CsA and tacrolimus has been studied in mice with controversial results [44] while everolimus has been shown to enhance the number of peripheral T-reg cells in liver recipients following a tacrolimus to everolimus conversion [48]. However, it was recently observed that sirolimus, another mTor inhibitor, did not expand peripheral T-reg cells in a cohort of de novo kidney recipients [49], in analogy to our present observation. Finally, in the present study we could not assess the possible role of lympho-depleting strategies, since induction treatment is not performed at our center.

We must acknowledge a number of limitations. First, although larger than other cohorts, our sample is not sufficiently large to identify small associations, especially in some subgroups. Second, patients started FU at different times from Tx; although this was taken into account in the analysis, we cannot be certain that bias would not impact on results. Third, although data were collected prospectively, this study was designed retrospectively as an exploratory analysis; therefore, patients were sampled at irregularly spaced times, and sometimes upon clinical basis, so there might be unobserved variables that might have affected results. In addition, we performed all the analysis using both the absolute number and the percentage of T-reg in peripheral blood, but, although both had the same trends over time and were significantly correlated with CLAD, we decided to show only the results concerning absolute number analysis because they had the best correlation with the analyzed variables. Finally, a relevant issue, from the biological point of view, is that graft tolerance is a results of a balance between regulatory and effector clones. In this study we couldn’t take into account the weight of allospecific effectors at different time points.

## Conclusion

In conclusion, given all the above limitations, we could find an association between T-reg cell counts and CLAD onset/progression, including both BOS and RAS phenotype, thus confirming animal observations on the protective role of T-reg cells with respect to CLAD.

## Abbreviations

AR: Acute rejection
BAL: Bronchoalveolar lavage
BOS: Bronchiolitis obliterans syndrome
CLAD: Chronic lung allograft dysfunction
CsA: Cyclosporine A
CT: Computed tomography
ECP: Extracorporeal photopheresis
FEV1: Forced expiratory volume in 1 second
FU: Follow-up
GER: Gastro esophageal reflux
IS: Immunosuppressant
LTx: Lung transplant
RAS: Restrictive allograft syndrome
T-reg: T regulatory cells
Tx: Transplant
